# Risk factors associated with Avian Influenza subtype H9 outbreaks in poultry farms in Kathmandu valley, Nepal

**DOI:** 10.1371/journal.pone.0223550

**Published:** 2020-04-02

**Authors:** Tulsi Ram Gompo, Bikas Raj Shah, Surendra Karki, Pragya Koirala, Manju Maharjan, Diker Dev Bhatt

**Affiliations:** 1 Department of Livestock Services, Central Veterinary Laboratory, Kathmandu, Nepal; 2 Institute of Agriculture and Animal Science, Tribhuvan University, Kathmandu, Nepal; 3 Himalayan College of Agricultural Sciences and Technology, Kathmandu, Nepal; Wageningen Universiteit en Researchcentrum IMARES, NETHERLANDS

## Abstract

The poultry sector contributes four percent to the national GDP of Nepal. However, this sector is under threat with periodic outbreaks of Avian Influenza (AI) subtypes H5 and H9 since 2009. This has been both a public health threat and an economic issue. Since the past few years, outbreaks of AI subtype H9 have caused huge economic losses in major poultry producing areas of Nepal. However, the risk factors associated with these outbreaks have not been assessed. A retrospective case-control study was conducted from April 2018 to May 2019 to understand the risk factors associated with AI subtype H9 outbreaks in Kathmandu valley. Out of 100 farms selected, 50 were “case” farms, confirmed positive to H9 at Central Veterinary Laboratory, Kathmandu, and another 50 farms were “control” farms, matched for farm size and locality within a radius of three km from the case farm. Each farm was visited to collect information using a semi-structured questionnaire. Twelve potential risk factors were included in the questionnaire under the broad categories: birds and farm characteristics, and management and biosecurity status of the farms. Univariable and multivariable logistic regression analysis was conducted and corresponding odds ratios were calculated. Risk factors, associated with AI subtype H9 outbreaks in Kathmandu valley, identified in the final multivariable model were: “farms that have flock size greater than median flock size of study farms (>1500)” (OR = 4.41, 95% CI: 1.53–12.71, p = 0.006), “farms that did not apply rules to wear boots for visitors inside the farms” (OR = 4.32, 95% CI: 1.52–12.29, p = 0.006) and “other commercial farms located within one km periphery” (OR = 10, 95% CI: 1.8–50, p = 0.007). This study showed that outbreaks of AI subtype H9 in Kathmandu valley were associated with a higher population of birds in the farm, poor management practices, and weak biosecurity measures in poultry farms. We suggest improving management practices and increase biosecurity in the farms to reduce incidences of AI subtype H9 outbreaks in Kathmandu valley.

## Introduction

Avian Influenza viruses (AIV) type A, belongs to *Orthomyxoviridae* family that can infect a wide range of species, though their known natural hosts are aquatic and wild birds [1–2]. AIV type A strains are broadly classified into two categories based on their pathogenicity: highly pathogenic avian influenza (HPAI), that causes severe illness and high mortality, and low pathogenic avian influenza (LPAI) that typically causes a mild illness with less severe or no clinical signs in birds [3]. Generally, HPAI is caused by AIV subtypes H5 or H7 but not all H5 and H7 are highly pathogenic [3]. HPAI has a zoonotic potential and can be transmitted to human from infected birds [4]. On the other hand, AI subtype H9 is generally, but not always LPAI, which is endemic in the poultry population of Eurasia and Africa [5]. The subtype H9N2 circulating in the Eurasian region has caused huge economic losses to the poultry industry, owing to a decline in egg production and high mortality when associated with other infections [6]. Also, as this virus has human-like receptor specificity [7], it possesses the potential to transmit to humans, posing a public health threat [8] . Moreover, it has been recognized recently that AI subtype H9 had shared gene segments to the highly zoonotic virus such as H7N9 that might contribute in the emergence of next influenza pandemic [9–10].

Nepal is an agrarian-based economy and the livestock sector including fisheries contributes nearly 12.5% to the total GDP. Among the livestock sub-sector, poultry alone contributes nearly four percent to the GDP [11]. The total population of poultry birds in Nepal is estimated to be nearly 72 million [12]. During the last three decades, the poultry industry globally has undergone rapid changes and has shifted towards intensive production systems, enhanced biosecurity, introductions of commercial breeds and application of preventive health measures [13]. While in developing countries like Nepal, these adoptions are limited due to high infrastructure costs for maintenance of biosecurity, quality hybrid chicks, qualitative feed, biologicals and quality veterinary care [14].

The booming poultry industry of Nepal has been hit by periodic outbreaks of avian influenza creating a great loss to the poultry industry. Nepal recorded the first HPAI outbreak in the eastern part of Nepal, Jhapa on January 16, 2009, where 28,000 poultry were killed to control the disease [15]. In the same year, the laboratory confirmed the first case of AI type A subtype H9 was reported from backyard poultry of Kathmandu although the first serological evidence of H9 was documented as early as 2005 by C-ELISA [16]. Thereafter, Nepal experienced several outbreaks of avian influenza, both H5 and H9, in consecutive years from 2010 to 2013 and from 2017 to 2019 [17]. Even now, there is a regular outbreak of avian influenza including AI subtype H9 in poultry that leads to the death of birds and production loss [18,19]. Out of total 1296 suspected clinical samples submitted to Central Veterinary Laboratory (CVL) for the five consecutive years 2013 to 2018 and tested by Real-Time Reverse Transcriptase Polymerase Chain Reaction (rRT-PCR), 42% (544/1296) were tested positive to H9. Beside the AI suspected samples, CVL received samples from pooled environmental and swab samples from poultry farms categorized as high risk districts by Government of Nepal as a part of the regular surveillance program with support from the Food and Agricultural Organization (FAO) [18, 20]. Out of 3930 cloacal and tracheal swab samples collected for bio-surveillance from August 2016 to July 2017, 0.41% (16/3930) samples were positive for H9. Likewise, out of 1597 swab samples collected for bio-surveillance from August 2017 to July 2018, 6.9% (110/1597) were tested positive for H9. The molecular tests performed on samples submitted from Nepal at OIE reference lab, Australian Animal Health Lab (AAHAL), Australia identified H5N1 virus to be of clade 2.3.2.1a and H9N2 to be of G1-like H9N2 lineage with closest the relationship to other G1-like H9N2 viruses that circulate in the South Asian region [18].

The first National Contingency Plan for Avian Influenza (HPAI) was drafted in 2003 (2060 B.S.) in Nepal, which only describes the contingency plans for HPAI but it does not spell out the provisions for control and containment of LPAI including H9 [21]. Kathmandu valley (Kathmandu, Bhaktapur and Lalitpur ), the capital of Nepal, has been identified as a high risk area for both LPAI and HPAI [22].There have been several outbreaks of AI subtype H5 and H9 in Kathmandu valley since 2013 [23], that caused massive economic losses and a direct negative effect on the livelihood of the farmers. In addition, the first human death case of AI subtype H5N1 was confirmed in Nepal in May 2019 [24]. Though AI subtype H9 outbreaks have increased in the last few years, limited studies have been conducted to investigate the causes associated with these outbreaks. The identification of the potential risk factors would be helpful to mitigate the disease outbreaks in the future. The objective of this study is to identify the risk factors associated with AI subtype H9 outbreaks in Kathmandu valley.

## Materials and methods

### Case definition and control farm selection

A retrospective case-control design was used in this study. The case registry book of Central Veterinary Laboratory (CVL), Tripureswor, Kathmandu was accessed from March 2018 to April 2019 for the study. A farm was considered as a case if it was confirmed positive for AI subtype H9 in rapid antigen detection test followed by Polymerase Chain Reaction (PCR). The control farms were any farms proximity to case farms (≤3 km from case farms) with no history of AI subtype H9 outbreak and confirmed negative to AI H9 by PCR during the time of the outbreak. Control farms were matched for farm size with case farms.

The sample size is determined with help of online epidemiological software “Epitools” [25] with desired power of 80%, assumed odds ratios of “3” and with expected proportion exposed in the controls is 40% at 95% confidence interval that produces 52 farms each. We initially selected 52 farms each but two farms were excluded from case and control farms as they did not fulfill the inclusion criteria.

Data were collected using a structured questionnaire having nineteen objective and open-ended questions. The questionnaire was pre-tested at ten farms of Kathmandu valley for its validity. Two veterinarians were trained to administer the questionnaire by face-to-face interview method. All the questionnaires were written in English, but the interview was conducted in local Nepali language. Written consent was obtained from each farm owner and kept their individual identity anonymous during the result publications.

To reduce the information and response bias, the responders were matched according to their duration of poultry farming and their education status. Only farmers who had reared poultry for more than 2 years and at least completed primary education were included in the study [26]. The majority of the poultry farmers and owners were aware of the bird flu as they were well educated from the mass media like radio and television [27].

The preliminary interview was conducted with poultry owners who came for the diagnostic services at CVL and subsequent farm visits were made to get detailed farm information.

Risk factors for avian influenza included in the questionnaire were identified from the literature review and expert’s opinion [28, 29]. The risk factors selected were divided into following two broad categories: i) Farm and bird characteristics ii) Management and biosecurity situation of the farm. In the farm and bird characteristic category, we documented the age of birds, median flock size of the farm, types of birds present on farms, distance from the nearest commercial farm and the farm distance from the main road were included as the potential risk factors [28, 29]. In the farm management and biosecurity category, the variable documented were culling practices of sick birds, flooring type of farm, previous history of AI (H9) outbreak on the farm, use of apron during farm operation, boots applied during farm operation and visitors allowed at farm.

### Site of study

**Fig 1:**
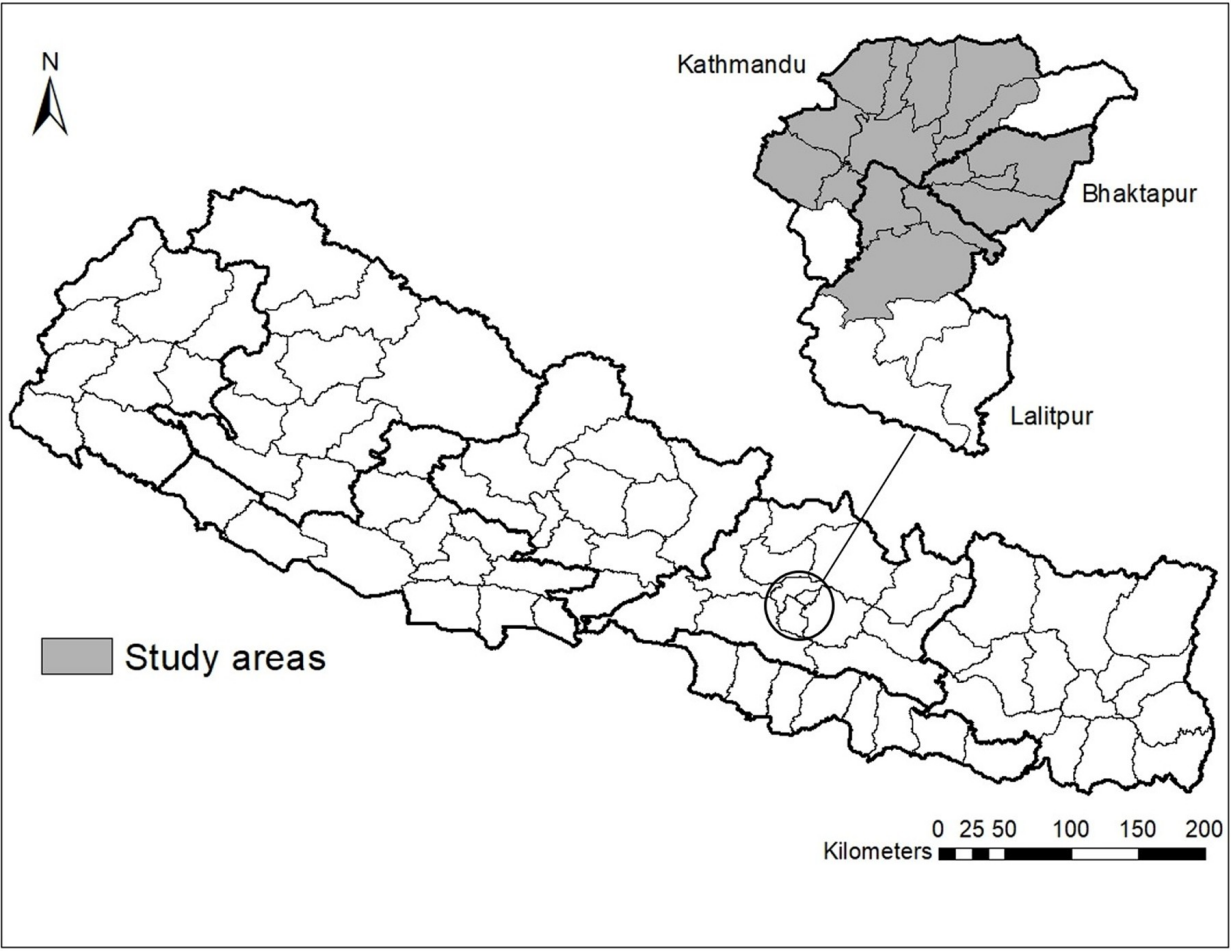
A map of Nepal with highlighted study sites.

The study was conducted in the poultry farms of Kathmandu valley. Kathmandu valley consists of three districts including capital the city, Kathmandu and adjacent districts Lalitpur and Bhaktapur with a total number of H9 outbreaks during the year April 2018 to April 2019 (Fig 1).

### Statistical analysis

Data were entered in Microsoft Excel 2016 and converted to CSV file for risk factor analysis in STATA 14.2. The continuous variables such as “age of birds in days”, “flock size”, “farm distance from the nearest commercial farms” and “farm distance from the main road” were tested individually to see normality in data distributions and q-q plot to see the relationship among them. All the four numerical variables “distance from the nearest commercial farm”, “farm distance from the main road”, “age of birds in days” and “flock size” were tested for multicollinearity. All the variables showed the non-linear relationships to one another. So, these continuous variables were transfigured into binary categorical variables using quantiles such as medians to avoid the problem of linearity [30]. The ages of birds are categorized into two groups (≤35 days and >35 days) with a median age of 35 days as cut off. The flock size is categorized into two groups with a median flock size of 1500 as a cut off (≤1500 and >1500) of the total studied poultry farms of Kathmandu valley. Any nearby commercial farm with 1 km from nearby commercial farms and farm located at a distance of 500 meters from the main road was considered a risk factor [28].

The 2×2 table analysis and chi-square test were performed to test independence between two categorical variables using online software OpenEpi version 3.01 and corresponding p-values were calculated. The groups of variables were tested for collinearity using Spearman rank correlation using the spearman functions in the STATA with cut off r≤0.5. None of the variables were found correlated with one another.

Univariable logistic regression analysis was applied to test the association of individual risk factors with the detection of AI subtype H9. Odds ratios (ORs), their 95% confidence intervals (CIs) and corresponding p-values were estimated by logistic functions in STATA.

Variables that met a cut-off of p≤ 0.15 in the univariable logistic regression were considered for the final multivariable logistic regression.

The models were built by both automated stepwise forward or automated stepwise backward in at a specified alfa level (p < 0.05) STATA [31]. The adjusted odds ratios from the multivariable regression were calculated to measure the strength of associations of the risk factors to detect AI subtype H9 in poultry farms of Kathmandu valley. The fitness of the final multivariable model was evaluated using the “estat gof” functions of Hosmer-Lemeshow test [32] in STATA (p = 0.6902).

## Results

### Population characteristics of poultry farms

**Fig 2:**
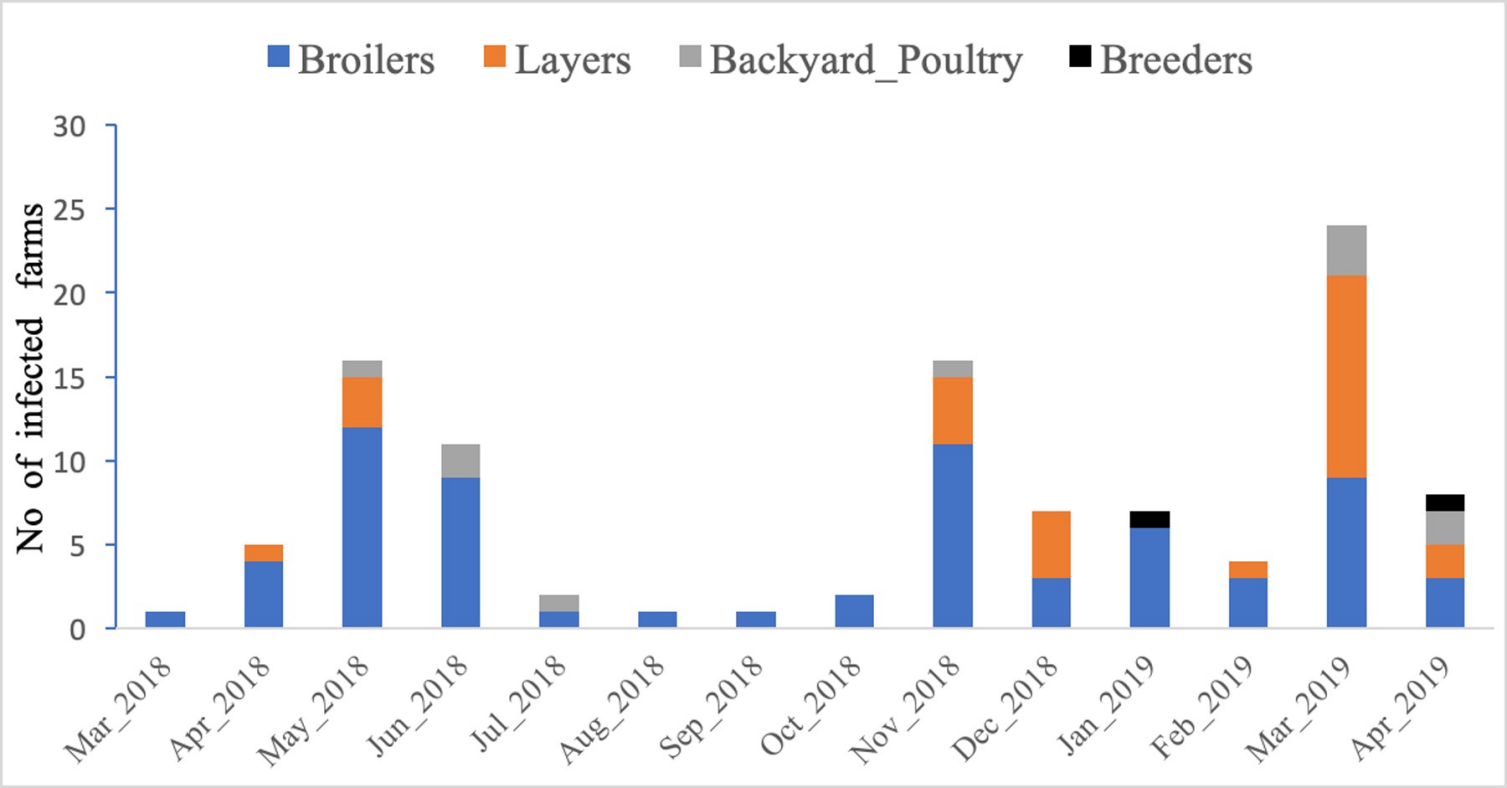
Epidemic curve for avian influenza subtype H9 infected farms in Kathmandu valley, Nepal.

The epidemic curve of AI subtype H9 outbreaks on farms of Kathmandu valley from March 2018 to April 2019 is shown in Fig 2. There were altogether 105 farms detected positive to AI subtype H9 during the study period in Kathmandu Valley. An outbreak started in March 2018 and the highest number of cases were observed in May 2018 with 16 farms infected which gradually decreased to one case farm in September 2018. Again, in November 2018, the number of infected farms rose to 16 and the outbreaks continued until January 2019. Later in March 2019, the outbreaks boomed to 24 and on average, eight farms remained infected until April 2019. Altogether 76 (61.9%) commercial broilers, 30 (24.4%) layers, 14 (11.4%) backyard poultry (local chicken and duck) and three (2.44%) breeder farms were confirmed positive to H9 by PCR at the period of study. The mean mortality percentage of birds in the studied population is 21.17% (95%CI: 16.38, 25.93). The median age of birds for the studied farms was 35 days. The mean flock size of the studied farms was 2003 (95% CI: 1578.144, 2429.23) and the median farm size was 1700 (range: 12–15000). Also, both the variables “age of birds in days” and “flock size” showed very weak correlations (r = 0.0284). There is a poor correlation between variables “farm distance from the nearest commercial farms” and “farm distance from the main road” (r = 0.1).

### Univariable analysis of risk factors

We selected total eleven risk factors and classified into two broad categories “bird and farm characteristics” and “farm management and biosecurity status”. Among them, only variables were significantly associated with H9 outbreak (p<0.05) by the univariable analysis. Under the bird and farm characteristics category: out of five variables tested, two variables: flock size of greater than 1500 (OR = 2.25, 95% CI: 1.01–5.01, p = 0.047) and farms located within a km from the nearest commercial farm (OR = 9.3, 95% CI: 1.9–43.7, p = 0.001) were significantly associated with the detection of AI subtype H9 (Table 1).

**Table 1:** Univariable logistic regression analysis of risk factors related to bird and farm characteristics.

Variables	Category	No of cases (n = 50)	No of controls (n = 50)	OR	95% CI	P value
Age of birds	>35 days	26	24	1.17	0.54, 2.57	0.689
	≤35 days	24	260			
Median Flock size	>1500	30	20	2.25	(1.01, 5.01)	0.047*
	≤ 1500	20	30			
Types of birds present on farms	Commercial Layers	15	9	1.85	(0.70, 4.91)	0.22
	Backyard_poultry	8	11	0.81	(0.28, 2.31)	0.69
	Commercial Broiler	27	30	Ref	-	-
Distance from the nearest commercial farm	≤1km	48	36	9.3	(1.9, 43.7)	0.005*
	>1km	2	14			
Farm distance from the main road	< = 500	39	35	1.519	(0.62,3.75)	0.37
	>500	11	15			

* P–value <0.05, statistically significant.

CI- Confidence interval; OR- Odds ratio.

Among the six variables under the farm management and biosecurity category, only two variables are border line significantly associated with the AI subtype H9 outbreak. They are “the previous history of AI outbreak” (OR = 7.98, 95%CI: 1.1–67.45, p = 0.03) and “No boots applied while entering farms “(OR = 2.4, 95% CI: 1.0–5.68, p = 0.05) (Table 2).

**Table 2:** Univariable logistic regression analysis of risk factors related to farm management and biosecurity status.

Variables	Category	No of case farms (n = 50)	No of control farms(n = 50)	OR	95% CI	P value
Culling of sick birds	Yes	35	36		-	-
	No	15	14	1.1	(0.46, 2.62)	0.83
Flooring type of farm	Muddy	38	36	1.23	(0.50, 3.02)	0.65
	Cemented	12	14			
Previous history of AI (H9) outbreak on the farm	Yes	7	1	7.98	(1.1, 67.45)	0.05*
	No	43	49			
Apron used during farm operation	Yes	21	26			
	No	29	24	1.5	(0.68, 3.29)	0.317
Boot applied during farm operation	Yes	30	39			
	No	20	11	2.4	(1.0, 5.68)	0.05*
Visitors allowed at the farm	Yes	24	17	1.80	(0.80, 4.01)	0.156
	No	26	33			

*P–value <0.05, statistically significant.

CI- Confidence interval; OR- Odds ratio.

### Multivariable logistic regression analysis

Based on the cut off criteria (p≤0.15), five variables that were significant at univariable analysis were included in the multivariable regression which, ultimately, produced four significant variables in the final model. Both the stepwise forward and backward model selection methods ended up with the same model (Table 3).

**Table 3:** Multivariable logistic regression of risk factors associated with Avian Influenza type H9 outbreaks on poultry farms in Kathmandu valley, Nepal.

Potential risk factors	Response level	Odds Ratio	95% CI for OR		p-value
Flock size of the farm	≤ 1500				
	>1500	2.55	(1.02,6.37)		0.044*
Distance from the nearest commercial farm	>1km				
	≤1km	10	(1.8, 50.0)		0.007*
Boots applied while entering farm	Yes				
	No	3.48	(1.3,12.29)		0.016*
History of previous outbreak of AI on the farm	Yes				
	No	.15	(.017,1.36)		0.091

* P–value <0.05, statistically significant.

CI- Confidence interval; OR- Odds ratio.

The farms that have median flock size of greater than >1500 birds, are almost three times more likely at risk of detecting AI subtype H9 (OR = 2.55, 95% CI: 1.02–6.37, p = 0.044) compared to farms that have median flock size of up to 1500.

The farms are 10 times more likely to be positive for AI subtype H9 when there are other commercial poultry farms within one km distance (OR = 10, 95% CI: 1.8–50.0, p = 0.007) compared to farms which do not have other commercial poultry farms within one km distance. The farms that did not applied rule to put on boots for anyone while entering the farms are fourfold at risk (OR = 3.48, 95% CI: 1.3–12.29, p = 0.016) compared to farms that applied the rules to use boots while entering the farms. (Table 3).

## Discussion

This is the first case-control study conducted to identify the risk factors associated with AI subtype H9 outbreaks in Nepal to the best of our knowledge. The farms that are located less than one km away from other commercial farms are more likely to be positive with the avian influenza H9 in Kathmandu valley. Other studies have also identified that the farms which are very close around 50 meters are more likely to be spread by wind [33] and there is a chance of mechanical transmission by vehicles as the delivery vans keep moving between the farms while delivering feeds and chicks between farms of the same area [34].

On the other hand, the farms where workers do not put on boots during the farm operations are at higher risk of being positive for AI subtype H9. This finding is consistent with the findings of Chaudhary et al., 2015, where “worker not change or disinfected boots” was found as a risk factor associated with the outbreak of AI subtype H9N2 in commercial poultry farms of Pakistan.

The farms that have birds number of greater than the median flock size of 1500 are more likely to be detected with AI subtype H9. It may be due to the compromised biosecurity in the farms due to higher bird transactions in bigger farms due to the regular selling and culling of birds.

## Limitations of the study

The farms that are close to CVL are more likely to submit samples than the farms located far away from the laboratory leading to selection bias. The farmers who are aware of AI and the diagnostic capability of the laboratory are more likely to visit the laboratory for the confirmation of the disease and small farms might have been missed. We could not interview many breeder farmers as they were not willing to share information as they were paranoid of the rejection of the chicks from their hatchery by the dealer if they know about their previous AI history.

## Conclusion

We identified risk factors related to poultry bird characteristics, farm management, and farm biosecurity characteristics that contribute to the outbreak of avian influenza AI subtype H9 in poultry farms of Kathmandu Valley. This study pinpoints that application simple biosecurity measures such as using separate boots in the farms could prevent in entry of AI subtype H9. The study also highlighted that consideration needs to be given while establishing a farm, as farms closer to other commercial farms will be at higher risk of contracting AI subtype H9. This study provides a baseline for similar studies in Nepal and other developing countries in the future.

### Recommendation

Good management and strict biosecurity can prevent AI subtype H9 infection in Kathmandu valley. The poultry farms should be established in isolated areas far from other commercial farms as far as practicable to prevent disease incursion. Any flock size greater than 2000 should follow stringent biosecurity practices to safeguard the farm from disease outbreak as farm-size was also identified as a risk factor for H9. Management of identified risk factors is a key consideration to mitigate the future risks of AI subtype H9 outbreak in Kathmandu valley. For this, a surveillance and contingency plan for AI subtype H9 should be prepared and implemented for effective control and containment of AI subtype H9 in Nepal. This will ultimately contribute to minimizing socioeconomic losses due to AI subtype H9 outbreaks. We suggest a detailed analytic study on this in the future taking more farms and other geographic areas of Nepal.

## Supporting information

S1 Appendix Questionnaire for “risk factors associated with AI subtype H9 outbreaks on poultry farms in Kathmandu valley, Nepal”. (PDF)
