# Supplementary material for: Risk factors associated with Avian Influenza subtype H9 outbreaks in poultry farms in Kathmandu valley, Nepal
Source: PLoS One. 2020 Apr 2;15(4):e0223550. doi: 10.1371/journal.pone.0223550 (PMC7117692; doi:10.1371/journal.pone.0223550)
Supplement: S1 Appendix — (PDF) [file pone.0223550.s001.pdf]

Technical Appendix S1

Questionnaire for “Risk factors associated with Avian Influenza subtype H9 outbreaks on poultry farms in Kathmandu valley, Nepal”.

This section must be filled in before completing the questionnaire:

I.D.: ………… Date: ………………………….

Name, address and location of poultry farmer: ………………………………

Contact No of poultry farmer: …………………………

Fill in the blank or put the number in given text box.

SECTION 1: Bird and farm characteristics

1. What is the total number of birds in your farm (Flock size)? …………………..
2. Can you tell me the of flocks/sheds in your farm premise? …………………..
3. What is the age of poultry you have on farm? ………………………………….
4. What is total percentage mortality after AI in the farm? ………………… ……..
5. What type of the poultry farm is it? ……… (1=Broiler; 2=Layer; 3=Duck; 4=Mixed; 5=Others.)
6. What is the age of the farm house? ………………
7. What type of farms are located nearby your farm? …… (1=Broiler; 2=Layer; 3=Duck; 4=Mixed; 5= Others)
8. What is the average distance of your farm from the main road? …………………

SECTION 2: Farm management

9. Do you do fumigation at farm after removal of birds in each batch? … (1=Yes, 2=No)
10. Do you cull of sick birds from the farm? …………. (1=Yes, 2=No)
11. What type of flooring material the farms has? ………. (1=Cemented 2=Muddy)
12. What is source of water supply to birds? ……. (1=Well, 2= Boring; 3=Tanker/ jar, 4=Stream/Spring)

13. Does your farm have previous history of outbreak of avian influenza (H9)? …… (1=Yes, 2=No)

SECTION 3: Biosecurity status

14. Do you use apron? ……… (1=Yes, 2 = No)
15. Do you use boots while entering farm? …………………… (1=Yes, 2=No)
16. Do you allow visitors to enter the farm? ……………………. (1=Yes, 2=No)
17. Do you apply self-sanitization before entering farm? ……… (1=Yes, 2=No)
18. Do you have foot bath at entry of farm? …………………….. (1=Yes, 2=No)
19. Does your farm has fencing around? ……………………….. (1=Yes, 2=No)
